# Adjuvant therapy for endometrial cancer in the era of molecular classification: radiotherapy, chemoradiation and novel targets for therapy

**DOI:** 10.1136/ijgc-2020-001822

**Published:** 2020-10-20

**Authors:** Anne Sophie V M van den Heerik, Nanda Horeweg, Stephanie M de Boer, Tjalling Bosse, Carien L Creutzberg

**Affiliations:** 1 Department of Radiation Oncology, Leiden University Medical Center Centrum, Leiden, Zuid-Holland, The Netherlands; 2 Department of Pathology, Leiden University Medical Center, Leiden, Zuid-Holland, The Netherlands

**Keywords:** endometrium, radiation oncology

## Abstract

Endometrial cancer is primarily treated with surgery. Adjuvant treatment strategies for endometrial cancer, such as external beam pelvic radiotherapy, vaginal brachytherapy, chemotherapy, and combined chemotherapy and radiotherapy, have been studied in several randomized trials. Adjuvant treatment is currently based on the presence of clinico-pathological risk factors. Low-risk disease is adequately managed with surgery alone. In high-intermediate risk endometrial cancer, adjuvant vaginal brachytherapy is recommended to maximize local control, with only mild side effects and without impact on quality of life. For high-risk endometrial cancer, recent large randomized trials support the use of pelvic radiotherapy, especially in stage I–II endometrial cancer with risk factors. For women with serous cancers and those with stage III disease, chemoradiation increased both recurrence-free and overall survival, while GOG-258 showed similar recurrence-free survival compared with six cycles of chemotherapy alone, but with better pelvic and para-aortic nodal control with combined chemotherapy and radiotherapy. Recent molecular studies, most notably the work from The Cancer Genome Atlas (TCGA) project, have shown that four endometrial cancer molecular classes can be distinguished; *POLE* ultra-mutated, microsatellite instable hypermutated, copy-number-low, and copy-number-high. Subsequent studies, using surrogate markers to identify groups analogous to TCGA sub-classes, showed that all four endometrial cancer sub-types are found across all stages, histological types, and grades. Moreover, the molecular sub-groups have proved to have a stronger prognostic impact than histo-pathological tumor characteristics. This introduces an new era of molecular classification based diagnostics and treatment approaches. Integration of the molecular factors and new therapeutic targets will lead to molecular-integrated adjuvant treatment including targeted treatments, which are the rationale of new and ongoing trials. This review presents an overview of current adjuvant treatment strategies in endometrial cancer, highlights the development and evaluation of a molecular-integrated risk profile, and briefly discusses ongoing developments in targeted treatment.

## Introduction

The majority of women with endometrial cancer are diagnosed with early-stage disease and have a favorable prognosis. Approximately 15–20% have an unfavorable prognosis with a high risk of distant metastases.^1^ The primary treatment of endometrial cancer is surgery, consisting of a total abdominal or laparoscopic hysterectomy and bilateral salpingo-oophorectomy. There is considerable controversy about whether lymphadenectomy should be part of standard care. Sentinel lymph node biopsy is increasingly used as an alternative for lymphadenectomy, as staging information can be obtained while sparing patients the morbidity of extensive lymph node dissection, especially lymphedema. The single-arm FIRES trial using indocyanine green to identify sentinel nodes, showed high sensitivity and negative predictive value of the sentinel lymph node procedure.^2^

Indications for adjuvant treatment have been primarily based on clinical and pathological factors, such as age, grade, histological type, depth of myometrial invasion, and presence of lymphovascular space invasion.^1^ Substantial lymphovascular space invasion is a strong prognostic factor for pelvic recurrence, distant metastasis, and decreased overall survival.^3^ Based on these prognostic factors, low, intermediate, high-intermediate, and high risk groups have been identified, each having a distinct prognosis and indications for adjuvant treatment (Table 1).^1 4 5^

**Table 1:** Different risk groups of endometrial carcinoma

Risk group	ESMO-ESGO-ESTRO consensus^1^	GOG-99^4^	PORTEC-1^5^
Low risk	Endometrioid endometrial cancer, grade 1–2, <50% myometrial invasion, lymphovascular space invasion negative	Endometrioid endometrial cancer, no myometrial invasion	Endometrioid endometrial cancer, any age, grade 1–2 <50% myometrial invasion
Low-intermediate risk	Endometrioid endometrial cancer, grade 1–2, ≥50% myometrial invasion, lymphovascular space invasion negative	Endometrioid endometrial cancer, not high-intermediate risk	Endometrioid endometrial cancer, grade 1–2, age <60, ≥50% myometrial invasion
High-intermediate risk	Endometrioid endometrial cancer, grade 3, <50% myometrial invasion, any lymphovascular space invasion	Endometrioid endometrial cancer, ≥50% with two factors: lymphovascular space invasion, grade 3, ≥66% myometrial invasion age or ≥70% with one factor	Endometrioid endometrial cancer, grade 1–2, age ≥60, ≥50% myometrial invasion
	Endometrioid endometrial cancer, grade 1–2, lymphovascular space invasion unequivocally positive, any myometrial invasion	Endometrioid endometrial cancer, any age, with all factors: grade 3, ≥66% myometrial invasion and lymphovascular space invasion	Endometrioid endometrial cancer, grade 3, age ≥60, <50% invasion
High	Endometrioid endometrial cancer, grade 3, ≥50% myometrial invasion, any lymphovascular space invasion	Stage II–III endometrioid endometrial cancer	Endometrioid endometrial cancer, grade 3, ≥50% myometrial invasion
	Stage II–III endometrioid endometrial cancer, no residual disease	Stage I–III non-endometrioid endometrial cancer	Stage II–III endometrioid endometrial cancer
	Non-endometrioid endometrial cancer stage I–III (serous, clear cell, or undifferentiated carcinosarcoma)		Stage I–III non-endometrioid endometrial cancer (serous or clear cell)
Advanced/ metastatic	Stage III with residual disease and stage IVa Stage IVb	Stage IV	Stage IV

ESGO, European Society of Gynecological Oncology; ESMO, European Society for Medical Oncology; ESTRO, European Society; GOG, Gynaecologic Oncology Group; PORTEC, Post Operative Radiation Therapy for Endometrial Carcinoma.

## Adjuvant Treatment in Endometrial Cancer

Multiple studies have assessed the role of radiotherapy, both external beam pelvic radiotherapy and vaginal brachytherapy, in the adjuvant treatment of women with endometrial cancer (Table 2).^4–8^ More recent trials focused on high-intermediate and high-risk disease, as there is currently no indication for adjuvant treatment in low-risk endometrial cancer.^1^

**Table 2:** Adjuvant radiotherapy in stage I–II endometrial cancer

Trial	Enrollment	No. of patients	Surgery	Eligibility	Randomization	Loco-regional recurrence	Survival
GOG-99^4^	1987–1995	392	TH-BSO+LND	Stages IB/C; stage II (occult)	EBRT vs NAT	2 years: 3% vs 12% (p=0.007)	4 years: 86% vs 92% (p=0.0557)
PORTEC-1^5^	1990–1997	714	TH-BSO	Stages IB G2-3; stages IC G1-2	EBRT vs NAT	5 years: 4% vs 14% (p<0.001)	5 years: 85% vs 81% (p=0.31)
Swedish^7^	1997–2008	527	TH-BSO	Stage I intermediate risk	VBT vs VBT+EBRT	5 years: 5% vs 1.5% (p=0.013)	5 years: 90% vs 89% (p=0.55)
ASTEC/EN.5^6^	1996–2008	905	TH-BSO±LND	Stages IA/B G3; IC; stage II; serous/CC	EBRT vs NAT	5 years: 6% vs 3% (p=0.02)	5 years: 84% vs 84% (p=0.98)
PORTEC-2^8^	2002–2006	427	TH-BSO	Age >60 and stage IB G3 or stages IC G1-2; stage IIA	EBRT vs VBT	5 years: 5% vs 2% (p=0.17)	5 years: 85% vs 80% (p=0.57)

LND; lymph node dissection G; grade; LND; lymph node G; grade; EBRT, external beam radiation therapy; GOG, Gynaecologic Oncology Group; NAT, no adjuvant treayment; PORTEC, Post Operative Radiation Therapy for Endometrial Carcinoma; TH-BSO, total hysterectomy and bilateral salpingo-oophorectomy; VBT, vaginal brachy therapy.

### Low-intermediate and High-intermediate Risk Endometrial Cancer

Randomized trials have shown that pelvic radiotherapy, compared with no additional treatment after surgery, significantly reduces loco-regional (vaginal and/or pelvic) relapse.^4–7^ However, pelvic radiotherapy does not lead to a decreased rate of distant metastasis or improved overall survival in early-stage disease. Moreover, patients treated with pelvic radiotherapy are at risk of toxicity, mainly gastrointestinal.^4–7^

In the observation arm of the randomized trials on the added value of adjuvant radiotherapy, most loco-regional relapses (75%) occurred in the vagina, particularly in the vaginal vault.^4 5^ Salvage treatment for vaginal relapse in patients who were not previously irradiated is effective.^9^ Among the patients in the control arm of the first Post Operative Radiation Therapy in Endometrial Carcinoma (PORTEC) trial who were treated for isolated vaginal relapse with pelvic radiotherapy and a vaginal brachytherapy boost, 89% had a complete remission and 3- and 5-year survival rates were 73% and 65%.^9^ In contrast, the prognosis of patients with pelvic or distant relapse was poor, with 3-year actuarial survival rates in the PORTEC-1 trial of 8% and 14%, respectively.^9^ These conclusions were confirmed in analysis of long-term outcomes.^10^ Based on these results it might be questioned if there is still an indication for adjuvant treatment in stage I endometrial cancer. However, in both the PORTEC-1 and Gynaecologic Oncology Group (GOG)−99 trials, about 30% of the patients were found to be at a relatively higher risk of recurrence without additional therapy. These patients were relatively older and had tumors with higher grade, deeper invasion, and presence of lymphovascular space invasion.^4 5^ This high-intermediate risk group had significant benefit from pelvic radiotherapy in terms of pelvic control; the 5-year risk of pelvic recurrence was reduced from 23% to 5%.^5 11^ In an analysis of long-term outcomes of the PORTEC-1 trial, it was found that patients with high-intermediate risk factors had about 20% risk of loco-regional recurrence with surgery alone, which was reduced to 5% with adjuvant pelvic radiotherapy.^10^ The results of the PORTEC-1 and GOG-99 trials led to a major decrease in the use of adjuvant radiotherapy, preventing the risk of radiotherapy-related morbidity for women with low-intermediate and intermediate risk endometrial cancer.

Since the vast majority of loco-regional recurrences were located in the vaginal vault, the PORTEC-2 trial was initiated to determine whether adjuvant vaginal brachytherapy would be as effective as pelvic radiotherapy in reducing vaginal recurrences in women with high-intermediate risk endometrial cancer.^8^ The 5-year results of the PORTEC-2 trial showed equally low rates of vaginal recurrence (1.8% for vaginal brachytherapy vs 1.6% for pelvic radiotherapy) in both treatment arms. No differences were found between the arms in disease-free and overall survival.^8^ In patients who received brachytherapy, lower rates of treatment-related toxicity and better health-related quality of life were observed. The health-related quality of life scores of women who received brachytherapy did not differ from those of an age-matched normative population.^8 12^ Similar results were found in a Swedish trial in which brachytherapy was compared with pelvic radiotherapy with a vaginal brachytherapy boost.^7^ Based on these results, adjuvant vaginal brachytherapy has become the standard of care in patients with high-intermediate risk endometrial cancer, ensuring maximal vaginal control without significant morbidity.^1^

### High-risk Endometrial Cancer

Approximately 15–20% of women with endometrial cancer are at increased risk of distant metastases and disease-related death, and are thus classified as high-risk.^1^ This comprises a heterogeneous group of histological types and stages, including endometrioid type stage I, grade 3 with deep myometrial invasion and/or substantial lymphovascular space invasion; stage II or III endometrioid endometrial cancer; and stage I–III non-endometrioid endometrial cancer (mainly serous or clear cell; Table 1). Non-endometrioid endometrial cancers have a higher risk of intra-abdominal or distant spread, which leads to a poorer prognosis. However, if diagnosed at an early stage, survival rates similar to grade 3 endometrioid endometrial cancer have been reported for both serous and clear cell endometrial cancer.^13^ Due to the heterogeneity of the high-risk patient population, adjuvant treatment strategies have been variable.

Historically, pelvic radiotherapy has been the standard adjuvant treatment to reduce the risk of pelvic recurrence in women with high-risk endometrial cancer. Multiple randomized trials have hypothesized that chemotherapy might improve survival by reducing the risk of metastatic disease. Initially, these trials focused on a comparison of adjuvant pelvic radiotherapy alone with chemotherapy alone. In the GOG-122 trial, whole-abdominal radiotherapy was compared with seven cycles of doxorubicin and cisplatin and one cycle with only cisplatin in women with stage III–IV disease, including those with residual disease <2 cm. Results of this trial showed that chemotherapy significantly improved overall survival, though at the cost of high toxicity rates and with high recurrence rates in both arms.^14^ Two randomized trials compared pelvic radiotherapy with adjuvant chemotherapy consisting of either three or five cycles of cyclophosphamide-doxorubicin-cisplatin, respectively, in stage I–III disease.^15 16^ Neither of these trials showed a difference in recurrence-free and overall survival. The Italian trial, in which most patients had stage III disease, seemed to indicate that chemotherapy delayed distant metastases, whereas radiotherapy delayed pelvic recurrence, with comparable recurrence-free and overall survival.^15^ In subsequent trials the combination of radiotherapy with chemotherapy (chemoradiation) has been assessed for its capacity to reduce both pelvic and distant recurrence (Table 3).

**Table 3:** Trials of adjuvant radiotherapy and chemotherapy in endometrial cancer

Trial	Enrollment	No. of patients	Eligibility	Randomization	5-Year overall survival	5-Year progression-free survival
Italian^15^	1990–1997	345	Stage I–II with grade 3 tumor; stage III	Pelvic RT vs 5 x CAP	69% vs 66% (NS)	63% vs 63% (NS)
GOG-122^14^	1992–2000	396	Stage III and IV, up to 2 cm residual disease after surgery allowed	Whole abdomen irradiation vs 8 x AP	42% vs 55% (p<0.01)	38% vs 50% (p<0.01)
Japanese^16^	1994–2000	385	Stage I–II with >50% myometrial invasion	Pelvic RT vs 3 x CAP	85% vs 87% (NS)	84% vs 82% (NS)
NSGO/EORTC pooled with Iliade-III^17^	1996–2007	534, NSGO/EORTC 378 and Iliade 156	NSGO/EORTC stage I–III; Iliade stage II–III	Pelvic RT vs pelvic RT and 4 x AP or TAP or TC or TEP	75% vs 82% (p=0.07)	69% vs 78% (p=0.02)
PORTEC-3^20^	2006–2013	686	Stage I–II with high-risk factors, stage III	Pelvic RT vs pelvic RT with 2 x CP followed by 4 x TC	76% vs 81% (p=0.034) Stage III 69% vs 79% Serous EC 53% vs 71%	69% vs 77% (p=0.016) Stage III 58% vs 71% Serous EC 47% vs 60%
GOG-249^18^	2009–2013	601	Stage I–II with high-intermediate or high-risk factors	Pelvic RT vs VBT and 3 x TC	87% vs 85% (NS)	76% vs 76% (NS)
GOG-258^21^	2009–2014	736	Stage III and IVa without residual disease up to 2 cm	Pelvic RT with 2 x CP followed by 4 x TC vs 6 x TC	70% vs 73% (NS)	59% vs 58% (NS)

AP, doxorubicin plus cisplatin; CAP, cyclophosphamide, doxorubicin, and cisplatin; CP, cisplatin; EC, endometrial cancer; GOG, Gynaecologic Oncology Group; NS, not significant; NSGO/EORTC, Nordic Society of Gynecologic Oncology/European Organization for Research and Treatment of Cancer; PORTEC, Post Operative Radiation Therapy for Endometrial Carcinoma; RT, radiation therapy; TAP, doxorubicin, cisplatin, and paclitaxel; TC, paclitaxel plus carboplatin; TEP, paclitaxel, epirubicin, and cisplatin; VBT, vaginal brachytherapy.

One of the first trials to report a favorable result with chemoradiation was the Nordic Society of Gynaecologic Oncology (NSGO)−9501/European Organization for Research and Treatment of Cancer (EORTC)−5591-trial.^17^ This trial randomly assigned women with mostly stage I endometrial cancer with grade 3 and/or deep invasion, to either radiotherapy with four cycles of platinum-based chemotherapy (doxorubicin or epirubicin with cisplatin or carboplatin plus paclitaxel or triplet therapy) or radiotherapy alone. The results were published in a pooled analysis with the unfinished ManGO Iliade-III trial. This Italian trial included women with more advanced stage disease (stage II–III) and compared pelvic radiotherapy with or without three cycles of doxorubicin and cisplatin. In the pooled population of 534 patients, chemoradiation yielded a significant improvement in recurrence-free survival and a trend for improved overall survival.^17^ In the more recent GOG-249 trial, 601 women with stage I–II endometrial cancer with high-intermediate or high-risk factors were randomly allocated to pelvic radiotherapy or brachytherapy and three cycles of carboplatin–paclitaxel chemotherapy. No differences in recurrence-free and overall survival were found.^18^ However, pelvic and para-aortic recurrences were significantly more frequent after brachytherapy and chemotherapy.^18^ The international PORTEC-3 trial included 660 evaluable patients with high-risk endometrial cancer (stage I, grade 3 with deep invasion and/or lymphovascular space invasion; stage II or III, or stage I–III with serous or clear cell histology) and compared pelvic radiotherapy alone with pelvic radiotherapy with two concurrent cycles of cisplatin followed by four cycles of adjuvant carboplatin and paclitaxel at 3-week intervals, based on a previous phase II Radiation Therapy Oncology Group (RTOG) trial.^19 20^ An overall survival benefit of 5% was observed with adjuvant chemotherapy.^20^ Among women with stage III disease and for those with serous cancers, a substantial improvement in both overall (of 10% for stage III and 18% for serous cancers, respectively) and failure-free survival (both 13%) was observed after combined chemoradiation.^20^

In the GOG-258 trial, 736 evaluable women with more advanced disease (stage III–IVa with or without residual disease up to 2 cm) were randomly assigned to pelvic radiotherapy with concurrent and adjuvant chemotherapy (using the same schedule as the PORTEC-3 trial) or chemotherapy alone (six cycles of carboplatin and paclitaxel). No significant differences were found in recurrence-free and overall survival.^21^ However, significantly higher rates of pelvic and para-aortic nodal recurrence (11% vs 20%, HR=0.43; 95% CI, 0.28 to 0.66) were seen in the chemotherapy-only arm.^21^

The use of adjuvant chemotherapy results in significantly more severe treatment-related morbidity. In the PORTEC-3 trial, grade ≥3 toxicities were significantly more common during and after treatment in the chemoradiation group (60% vs 12%); which were mainly hematological, gastrointestinal, bone, joint, and muscle-related adverse events.^22^ Patients recovered well in the first year after completion of treatment, although still more grade 2 toxicity, mainly sensory neuropathy, was observed in the chemoradiation arm at 3 years. About 25% of the women in the chemoradiation arm reported ‘quite a bit’ or ‘very much’ tingling or numbness.^23^ These results are consistent with reports of toxicity and quality of life from the GOG-249 trial.^18^

Based on these recent trials it can be concluded that current evidence supports the use of chemoradiation to maximize recurrence-free and overall survival, as well as pelvic and para-aortic nodal control in women with stage III disease and/or serous histology. Still, there is debate among physicians as to whether the combined chemoradiation schedule should be preferred over chemotherapy alone, in view of similar relapse-free survival rates in the GOG-258 trial and concerns about suggested higher toxicity. However, significantly more vaginal, pelvic, and para-aortic nodal recurrences were reported in the chemotherapy alone arm, and it is not reported how many patients in the chemotherapy alone arm received radiotherapy at the time of relapse.^21^ Most severe toxicities in both the GOG-258 and PORTEC-3 trials were related to chemotherapy (especially hematological, joint, and muscle-related symptoms and sensory neuropathy).^23^

Although salvage rates of isolated vaginal recurrence are favorable, the prognosis of patients with a pelvic and/or para-aortic nodal recurrence, especially with high-grade/advanced disease remains poor, both due to lower control rates and higher risk of subsequent distant metastases.^9 24^ More recent small case series reporting on intensity-modulated radiation therapy with or without chemotherapy for nodal relapse in patients who had no previous radiotherapy showed 2-year overall survival rates of around 70% with acceptable rates of toxicity.^25–27^ An ongoing GOG trial (ClinicalTrials.gov Identifier NCT00492778) evaluates the benefit of concurrent cisplatin with radiation therapy for women with pelvic and/or vaginal recurrences.

In the past two decades, conventional pelvic radiation using 3D conformal four-field techniques have increasingly been replaced by intensity-modulated radiation therapy and volumetric arc techniques. Both techniques are associated with reduced doses to the organs at risk, resulting in a reduction of radiation related toxicities. The RTOG 1203 trial was the first randomized trial to compare intensity-modulated radiation therapy with 3D conformal techniques in relation to acute patient-reported toxicity, and showed that significantly less gastrointestinal and urinary morbidity was reported by patients in the intensity-modulated radiation therapy arm.^28 29^

The optimal sequencing of chemotherapy and radiotherapy is a subject of controversy. Many centers prefer sequential treatment, giving chemotherapy first, which is often based on logistical reasons and on the principle that chemotherapy should be initiated early to treat occult distant metastases. In the pooled MaNGO/Iliade trials, which used either sequence of therapy (majority of patients having received chemotherapy first), no difference in outcomes was seen between patients who first received chemotherapy and those who first received radiotherapy.^17^ Some centers prefer so-called sandwich therapy (chemotherapy followed by radiation followed by chemotherapy again), which seemed to improve 3-year outcomes in a multicenter retrospective analysis.^30^ However, published results from two recent large prospective randomized trials have shown the safety and activity of the combined chemotherapy and radiotherapy schedule, which was based on the RTOG phase II trial. This schedule has the advantage of starting both adjuvant treatments immediately after surgery, and has a shorter overall treatment duration than when sequencing therapy.

There is a paucity of data on adjuvant treatment for stage IA serous and clear cell cancers without myometrial invasion and/or occurring in a polyp. Case series have shown that these patients have a favorable prognosis. Based on a cohort of 103 patients, including 87 with stage IA disease (27 non-invasive) treated at the Mayo Clinic who were fully surgically staged, it was concluded that vaginal brachytherapy would be the adjuvant treatment of choice, whereas additional chemotherapy did not seem to improve outcomes.^31^ However, in a recent retrospective analysis from the National Cancer Database on 1709 patients with serous cancers confined to the myometrium, 5-year overall survival was 82% for those who had no adjuvant therapy, compared with 85% for those who received adjuvant radiotherapy, and 91% for those who received adjuvant chemoradiation or chemotherapy alone. In view of all of the biases associated with these retrospective analyses, the conclusion was that that we do not yet have sufficient data to uniformly recommend chemotherapy for stage I non-invasive serous endometrial cancer.^31 32^

## Pathology Evaluation

Pathology of the female reproductive tract is known to have high rates of inter-observer variability, especially in assessment of endometrial cancer, which could have consequences for adjuvant treatment selection.^33–35^ Multiple studies have shown inter-observer variations in assessment of grade and histological type of endometrial cancer, but also in the determination of endocervical involvement and lymphovascular space invasion.^11 33–35^

The use of a binary grading system differentiating between low-grade (grade 1–2) and high-grade (grade 3) disease and the addition of immunohistochemistry for determination of histological type have been suggested to improve inter-observer agreement and have more prognostic value.^35 36^ Assessment of lymphovascular space invasion is more robust if substantial lymphovascular space invasion is differentiated from no or focal lymphovascular space invasion.^3^ The three-tiered semi-quantified scoring system (no, focal or substantial lymphovascular space invasion) has proved to be the most powerful and to lead to the least inter-observer discordance.^3 37^ In this three-tiered system focal lymphovascular space invasion is defined as a single focus of lymphovascular space invasion around a tumor, whereas substantial lymphovascular space invasion is defined as diffuse or multifocal lymphovascular space invasion around a tumor. Difficulties in differentiating between focal and substantial can arise in cases with two to five involved vessels, as more than four vessels is usually regarded as extensive. Other analyses have used ‘unequivocal’ or ‘obvious’ to refer to non-focal lymphovascular space invasion. Even though it is difficult to determine a precise cut-off value, it is clear that lymphovascular space invasion should be non-focal and clear to the pathologist to be of strong prognostic significance.^1 37 38^

In the PORTEC-3 trial, a pathology review was performed prior to randomization to ensure inclusion of only true high-risk patients. At pathology review, 8% of patients did not fulfill the eligibility criteria, most often as a result of differences in the assessment of histological type, endocervical stromal involvement, and histological grade.^35^ This percentage of discordant pathology is in line with previous studies, and these patients were not included in the PORTEC-3 trial.^34^

## Molecular Subgroups of Endometrial Cancer

### Four Molecular Subgroups as Defined by the Cancer Genome Atlas

The Cancer Genome Atlas (TCGA) project describes the molecular landscape of endometrial cancers (mainly endometrioid and serous histologies) by a comprehensive multi-omic analysis of 373 cases.^39^ TCGA identified four molecular sub-classes based on somatic mutational burden and copy number alterations: (i) ultra-mutated endometrial cancer with mutations in the exonuclease domain of *DNA polymerase epsilon* (*POLE*), (ii) hypermutated endometrial cancer with microsatellite instability, (iii) copy-number-high endometrial cancer with frequent *TP53* mutations, and (iv) the copy-number-low group of endometrial cancers. Differentiating between these four molecular sub-groups was shown to be of prognostic relevance.^39^

The ultra-mutated endometrial cancers are characterized by pathogenic variants in the exonuclease domain of *POLE*. These mutations in *POLE* result in proofreading dysfunction during DNA replication, leading to the exceptionally high mutational burden observed in these endometrial cancers. A limited number of pathogenic *POLE* variants within the exonuclease domain have been identified as resulting in an ultra-mutated phenotype in endometrial cancer.^40 41^ Approximately 8–10% of all endometrial cancers carry one of these pathogenic *POLE* mutations.^39^ In the molecular classification of endometrial cancer these cases are referred as *POLE*mut. Typically, these *POLE*mut endometrial cancers are found in relatively young women with early-stage but high-grade tumors with prominent lymphocytic infiltrate.^39 42 43^ Despite being high grade, *POLE*mut tumors are associated with an exceptionally favorable prognosis with only rare relapse regardless of adjuvant treatment.^39 44 45^ It is hypothesized that tumor neopeptides caused by ultra-mutation may elicit a strong cytolytic immune response.^42 43 46^ In addition, it has been speculated that the ultra-mutated status impairs functioning of *POLE*mut cancer cells, leading to reduced metastatic potential.

The microsatellite instable group is more often referred to as the group with mismatch repair deficiency, as immunohistochemistry has largely replaced detection of microsatellite instability. The mismatch repair deficiency endometrial cancer sub-group comprises about 25–30% of all endometrial cancers and is defined by the loss of nuclear expression of one or more mismatch repair proteins, leading to an accumulation of mismatches, insertions, and deletions.^39 44 47^ Mismatch repair deficiency is most often caused by somatic events such as *MLH1* promoter hypermethylation. In a small proportion of cases it is due a germline mutation in one of the mismatch repair genes, defined as Lynch syndrome.^48^ Mismatch repair deficiency endometrial cancer also elicits a strong immunogenic response and has an intermediate prognosis.^39 43 46^

The third molecular sub-group consists of tumors with a high number of somatic copy number alterations and a relatively low somatic mutation rate, but with frequent occurrence of *TP53* mutations, in 90% of the cases.^39^ This group comprises mainly high-grade cancers with a poor prognosis due to aggressive growth with early spread of disease. Non-endometrioid histologies, most typically serous cancer and carcinosarcoma, but also about 50% of clear cell cancers, dominate this molecular sub-group. However, (high-grade) endometrioid endometrial cancers with *TP53* mutations (which is found in about 61% of grade 3 endometrial cancers) are also included, which when molecularly classified have a similarly poor prognosis.^45 49^ Recent studies have shown frequent homologous recombination deficiency in p53 abnormal staining (p53abn) endometrial cancer.^50^

The fourth and largest sub-group of copy-number-low endometrial cancer, also referred to as endometrial cancer with no specific molecular profile, has both a low mutational burden and low number of somatic copy number alterations. Prognosis in these tumors is stage dependent, but together can be regarded as intermediate risk.^39^ This group typically contains endometrioid-type cancers with positive staining for estrogen and progesterone receptors. The molecular heterogeneity within this group suggests that further refinement in distinct sub-sets within this group may be possible. One candidate for prognostic refinement may be the presence of mutations in exon 3 of β-catenin (*CTNNB1*). These are identified in 30–50% of endometrial cancers in this sub-group, and the prognosis has been reported to be relatively poor compared with those no specific molecular profile endometrial cancers without *CTNNB1* mutations.^39 51^

**Figure 1:**
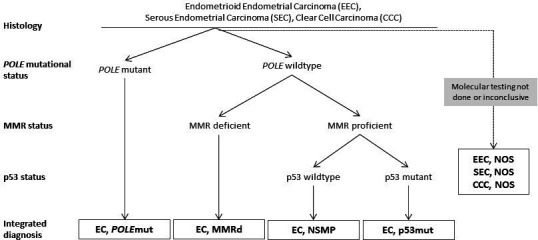
Diagnostic algorithm for the classification of the four molecular sub-groups in endometrial cancer. Reprinted from 'Incorporation of molecular characteristics into endometrial cancer management' by Vermij et al.^52^ EC, endometrial cancer; MMR; mismatch repair; MMRd, mismatch repair deficiency; NSMP, no specific molecular profile; NOS, not otherwise specified; POLEmut; POLE mutated.

Most endometrial cancers can directly be classified into one of the four molecular sub-groups using the surrogate marker approach (Figure 1).^52^ However, about 3–6% have more than one classifying alteration (for example, both p53 abnormal staining and a pathogenic *POLE*mut), and are referred to as multiple-classifier endometrial cancers. Recently, a comprehensive study evaluating histology, molecular landscape, and the clinical behavior of multiple-classifier cancers has shown that *TP53* mutations can occur as a secondary event in the context of the 'mutators' mismatch repair deficiency and *POLE*mut endometrial cancers, without affecting outcome. Evidence supports classifying endometrial cancers with a pathogenic *POLE* variant in the exonuclease domain as a *POLE*mut endometrial cancer, independent of the co-occurrence of mismatch repair deficiency or mutant-like abnormal p53 immunostaining.^53^

### Molecular-integrated Risk Profile

Both the PORTEC group and the Proactive Molecular Risk Classifier for Endometrial Cancer (PRoMisE) study group have classified the molecular sub-groups by their surrogate markers in paraffin-embedded tissues.^44 47^ In addition to TCGA molecular groups, several other clinico-pathologic and molecular risk factors have proved to be prognostic, such as substantial (diffuse or multifocal) lymphovascular space invasion, L1-cell adhesion molecule over-expression, *CTNNB1* mutation, and 1q32.1 amplification; the last two being mostly discriminative within the no specific molecular profile sub-group.^44 47 54^ L1-cell adhesion molecule is a membrane glycoprotein with an important role in tumor cell adhesion and migration. It is strongly associated with *TP53* mutations, non-endometrioid histology, high tumor grade, lymphovascular space invasion, and is an independent risk factor for loco-regional and distant spread.^55 56^ *CTNNB1* mutations result in growth stimulation of endometrial tissues, which is associated with higher risk of recurrence and decreased recurrence-free survival.^39 51^ Amplification of 1q32.1 has been reported to be associated with significantly worse prognosis in the no specific molecular profile sub-group.^54^

Comprehensive analysis of the prognostic significance of these risk factors with TGCA sub-groups as determined by their surrogate markers was done in a multivariate analysis using >800 stage I endometrial cancers from the combined PORTEC-1 and PORTEC-2 biobank.^44^ The resulting molecular-integrated risk profiles within the group of high-intermediate risk endometrial cancers were able to distinguish patients with a favorable profile (50% of patients), intermediate profile (35%), and unfavorable profile (15%), with a significantly different recurrence-free survival and a high diagnostic reproducibility.^44^ In the ProMisE validation studies it was also found that the highest prognostic significance was obtained when the molecular sub-groups were combined with the clinico-pathological risk factors.^56 57^

**Figure 2:**
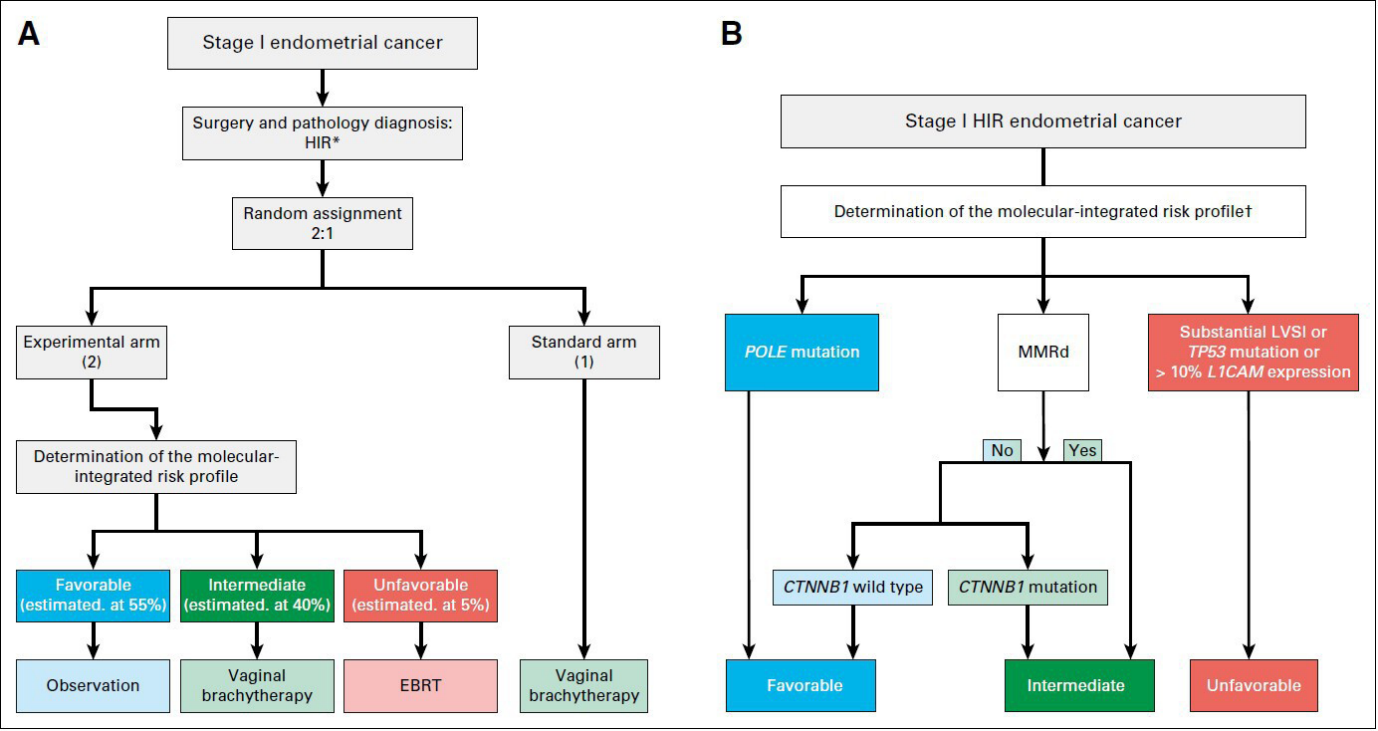
Study design of the PORTEC-4a trial. Reproduced with permissionfrom 'Molecular-integrated risk profile to determine adjuvant radiotherapy in endometrial cancer: evaluation of the pilot phase of the PORTEC-4a trial' by Wortman et al.^58^ CTNNB1, β-catenin; EBRT, external beam radiation therapy; HIR, high-intermediate risk; LVSI; lymphovascular space invasion; MMRd, mismatch repair deficiency.* High-intermediate risk endometrial cancer: stage IA (with invasion) and grade 3; stage IB, grade 1 or 2 with either age ≥60 or substantial lymphovascular space invasion; stage IB, grade 3 without lymphovascular space invasion; or stage II (microscopic) and grade 1.

The ongoing randomized PORTEC-4a trial (NCT03469674) is the first clinical trial to prospectively investigate the use of an integrated clinico-pathological and molecular risk profile for the selection of adjuvant therapy. In this trial, the four molecular sub-groups are combined with other prognostic factors (substantial lymphovascular space invasion, L1-cell adhesion molecule expression, and *CTNNB1* mutation) to designate favorable, intermediate, and unfavorable profile.^58^ Participating women with high-intermediate risk endometrial cancer are randomized (2:1) to adjuvant treatment based on their molecular-integrated risk profile or standard adjuvant vaginal brachytherapy. Patients in the intervention arm do not receive adjuvant treatment if they have a favorable profile, whereas those with an intermediate-risk profile receive standard adjuvant vaginal brachytherapy. Only the small proportion with an unfavorable profile receive pelvic radiotherapy (Figure 2). It is expected that the PORTEC-4a trial will provide essential data on decreasing both over-treatment and under-treatment by risk profile-based treatment selection for patients with high-intermediate risk endometrial cancer.

### Prognostic Relevance of Molecular Sub-groups in High-risk Endometrial Cancer

Results of PORTEC-3 related translational research on outcomes and differences between the trial arms by molecular sub-group were presented at international meetings and have been recently published.^45^ Even in these high-risk cancers the molecular groups were found across all histological sub-types, stages, and grades. Moreover, clear differences in prognosis between the molecular sub-groups were observed, as was found also in an international molecular analysis of grade 3 endometrial cancers.^59^ In the PORTEC-3 translational study, patients with p53abn endometrial cancer had the worst outcome, with a significant benefit of added adjuvant chemotherapy: 5-year recurrence-free survival was 59% with combined chemotherapy and radiotherapy versus 36% with radiotherapy alone (p=0.019). Similar unfavorable outcomes were found in both serous and non-serous histologies within the p53abn sub-group. Mismatch repair deficiency endometrial cancers had an intermediate prognosis, and 5-year recurrence-free survival was similar for radiotherapy and chemoradiation. Hence, adding chemotherapy to pelvic radiotherapy did not seem to reduce recurrence in high-risk mismatch repair deficiency endometrial cancer. Patients with *POLE*mut endometrial cancer had excellent outcomes with only one relapse in the radiotherapy group and 5-year recurrence-free survival of 100% with chemoradiation versus 97% with radiotherapy alone. The no specific molecular profile group had an intermediate outcome, with some benefit of added chemotherapy, similar to the overall PORTEC-3 trial results.^45^

## Targeted Therapy

Although patients with endometrial cancer in general have a favorable prognosis, for those who are diagnosed with recurrent or metastatic disease, overall survival remains poor.^1^ Standard systemic treatment for metastatic disease comprises hormonal therapy for those with low-grade, estrogen and progesterone receptor positive tumors, and chemotherapy (first-line therapy being carboplatin and paclitaxel) for all others.^1^ More recently, studies on the molecular characteristics of endometrial cancer have led to identification of targetable molecular alterations within all four molecular sub-groups, which have led to exploration of more individualized treatments based on agents directed against these molecular alterations. A large number of exploratory studies and early-phase clinical trials of targeted therapies have been published. The different types of targeted therapy are directed to (i) the immune system (checkpoint inhibitors), (ii) DNA repair mechanisms, and (iii) cellular pathways, and may be used as monotherapy or combination treatment.

### Checkpoint Inhibitors

Hypermutated tumors, such as *POLE*mut and mismatch repair deficiency endometrial cancers, are highly immunogenic due to their mutational burden and are associated with high levels of neoantigens and tumor-infiltrating CD8+ T cells.^43^ However, when the programmed death-ligand 1 (PD-L1) receptor on cancer cells interacts with programmed death protein 1 (PD-1) on activated T cells, the immunological response is suppressed and apoptosis is inhibited.^60 61^ Recently, the efficacy of checkpoint inhibitors, such as the PD-1 inhibitors (nivolumab, pembrolizumab) and PD-L1 inhibitors (atezolizumab, avelumab, durvalumab), both as monotherapy and in combination with chemotherapy, has been shown in various solid cancers, especially those with mismatch repair deficiency.^62^ A study on pembrolizumab, which included 49 patients with endometrial cancer with unresectable or metastatic mismatch repair deficiency tumors, showed an overall response rate of 57%.^63^ However, only eight of the 49 included patients achieved complete response and the median progression-free survival was 26 months.^63^ Similar results have been seen with the use of avelumab (overall response rate of 26.7%) and durvalumab (overall response rate of 43%) as monotherapy in mismatch repair deficiency advanced endometrial cancer.^64 65^

### DNA Damage Response Inhibitor

One of the key enzymes in DNA repair mechanisms is poly (ADP-ribose) polymerase (PARP). Usually, a double-strand DNA break is repaired by either homologous recombination repair or, in its absence, the more error prone non-homologous end joining. If a tumor is homologous recombination deficient, the DNA repair pathway is modulated by PARP.^61^ PARP inhibitors, such as olaparib, niraparib, rucaparib, talazoparib, and veliparib, impede DNA repair and lead to an accumulation of double-strand DNA breaks in the tumor cells. This results in genomic instability and eventually to cell cycle arrest or cell death. It is hypothesized that the homologous recombination deficient cancer cells become more sensitized to DNA-damaging agents if PARP inhibitors are administered, leading to synthetic lethality.^66^ In the copy-number-high subclass of endometrial cancer, homologous recombination deficiency has been observed frequently. In a small and selected set of cases, 46% of *TP53-*mutant endometrial cancers were reported to be homologous recombination deficient by functional RAD51 assessment.^50^ No data are yet available on the effect of PARP inhibition in patients with endometrial cancer, but combination treatment targeting homologous recombination deficiency using platinum-based chemotherapy with PARP inhibition or a combination of PARP and checkpoint inhibition seems attractive based on the similarities with high-grade ovarian cancer.^61^

### Cellular Pathway Inhibitors

The most frequent altered pathway in endometrial cancer is the phosphatidylinositol 3-kinase-AKT-mammalian target of rapamycin (PI3K/AKT/mTOR) pathway.^39^ This pathway regulates key aspects of cancer biology, including cell growth and survival.^67^ Dysregulation can occur by many different mechanisms, including the inactivation of *PTEN*, which is the primary negative regulator, or mutations in *PI3K3CA* and *KRAS*. Loss of *PTEN* expression is frequent in (low-grade) endometrioid-type endometrial cancer with favorable prognosis. *PI3K3CA* mutations are more frequently associated with higher tumor grade.^39^ This pathway has many potential alterations, and targeted therapies fall into four main categories: (i) mTOR inhibitors, (ii) PI3K inhibitors, (iii) dual mTOR/PI3K inhibitors. and (iv) AKT inhibitors. The majority of clinical studies have focused on mTOR inhibitors, either as single agent or in combined treatment, for their acceptable toxicity profile.^67^ Only modest results have been observed in studies using mTOR inhibitors alone.^68^ However, the combination of rapamycin analogs and an aromatase inhibitor showed favorable results.^68^ A response rate of 32% was seen with combined treatment of everolimus and letrozole in a phase II study among chemo-naïve patients with recurrent disease.^69^ Subsequently, the combination of everolimus, letrozole, and metformin was investigated, since prior research had demonstrated inhibition of proliferation and induction of apoptosis with metformin.^70^ A clinical benefit in half of the 54 patients with metastatic endometrioid endometrial cancer was seen, with a partial response in 28% and stable disease in 22% after 16 weeks of therapy. However, the addition of metformin to the combination did not appear to improve the outcomes.^70^

The human epidermal growth factor receptor HER2 provides critical signaling for cancer cell growth, survival, and proliferation.^39^ The majority of endometrial cancers, which over-express HER2 are mainly serous or *TP53*-mutated cancers, including carcinosarcomas. In TCGA analysis, HER2 was amplified in 25% of the copy-number-high/*TP53*-mutated endometrial cancers.^39^ No major responses were observed in phase II trials with trastuzumab, either as single agent or in combination with pertuzumab.^71^ Possible causes are inclusion of heavily previously treated patients, interaction of mutations in the PI3K pathway which could induce trastuzumab resistance, and increased expression of a constitutively active p95HER2 truncated variant, which lacks the trastuzumab binding domain.^72^ Based on these findings, the use of trastuzumab combined with chemotherapy was explored in a phase II trial among women with stage III–IV HER2-positive serous endometrial cancers. A prolonged median progression-free survival was observed; 9.3 months in the control versus 17.9 months in the experimental arm.^73^

### Combination Therapies

A phase II clinical trial presented interim results on combining immunotherapy with vascular endothelial growth factor and tyrosine kinase inhibition.^74^ Women with metastatic endometrial carcinoma, irrespective of mismatch repair deficiency or PD-L1 expression status, were treated with a combination of pembrolizumab and lenvatinib, a multikinase inhibitor. An objective response was found in 21 of the 54 enrolled participants, which indicates that this combination could be a new potential treatment option.^74^ Two randomized phase III trials (KEYNOTE-775/NCT03517449, ENGOT-EN9/LEAP-001/NCT03884101) on the combinational use of lenvatinib and pembrolizumab versus chemotherapy are currently recruiting.

Monotherapy by PARP inhibitors and PD-1/PD-L1 inhibitors have shown promising results.^61^ Several phase II trials are currently investigating whether combination therapy of PARP inhibition and PD-1/PD-L1 pathway inhibition are even more effective, as limited data have suggested an added or even synergistic effect.^75^ The NCT02912572 trial will include 70 patients with metastatic endometrial cancer who have previously been treated with at least one line of chemotherapy. The first cohort will include patients with mismatch repair deficiency and/or *POLE*mut endometrial cancer and treat them with avelumab monotherapy. The second cohort will include patients with microsatellite-stable tumors without a pathogenic *POLE* mutation and treat them with both avelumab and talazoparib. The DOMEC trial (NCT03951415) is an ongoing multicenter single-arm phase II trial that will include 55 patients with metastatic endometrial cancer (including carcinosarcoma) to investigate the response and recurrence-free survival with the combination of olaparib and durvalumab.

### Combining targeted therapy and radiation

Ionizing radiation induces genomic instability via both direct and indirect DNA damage, which produces free radicals that trigger chemical reactions that cause potentially lethal damage, leading to cellular death.^76^ Theoretically, combination of radiotherapy with PARP inhibitors leads to inhibition of DNA repair in the malignant cells, which contributes to DNA damage and apoptosis.^77^ Second, radiation enhances tumor immunogenicity through the release of pro-inflammatory cytokines and chemokines via uptake of tumor antigen and cross-presentation by dendritic cells.^78^ The rationale for combining immunotherapy and radiation is the alteration of the tumor microenvironment by radiation, which intensifies the recruitment and infiltration of immune cells. However, the optimal treatment schedules for timing and dose are still unclear and are a subject of further research.

## Conclusion and Future Perspectives

Adjuvant treatment for patients with endometrial cancer has become increasingly risk-based using clinico-pathologic risk factors. The molecular sub-groups of endometrial cancer provide the basis for a more robust classification of endometrial cancer with prognostic significance. Essential findings are that the molecular classification provides a prognostic model, and has also been shown to predict response to chemotherapy and to be associated with specific molecular targets against which targeted drugs are available. Especially studies using immune checkpoint inhibitors for patients with mismatch repair deficiency endometrial cancers have shown high rates of response, and recent studies of other targeted agents, including PARP inhibitors targeting homologous recombination deficiency and antibodies targeting HER2 over-expression within the p53abn group, have shown promising results. Recently, the results of molecular analysis of the PORTEC-3 tissue samples showed that even in these high-risk endometrial cancers, all four molecular sub-groups are found, with clear prognostic differences. Moreover, differences in benefit of chemoradiation as compared with radiotherapy alone were found between the molecular groups, with strong and significant benefit of added adjuvant chemotherapy in patients with p53 mutational expression, whereas those with *POLE* mutation had almost 100% recurrence-free survival in both arms. Mismatch repair deficiency cancers do not seem to benefit from added chemotherapy, whereas those with no specific molecular profile had slightly higher relapse-free survival with chemoradiation, comparable to the overall PORTEC-3 trial outcomes. Thus, better selection of adjuvant treatment can be achieved when treatment is based on molecular group, and targeted agents such as immune checkpoint inhibition for mismatch repair deficiency cancers might be more effective and results in less toxicity. A planned study is the comprehensive RAINBO trials program for patients with high-risk and advanced-stage endometrial cancer, selecting and comparing adjuvant treatments based on the molecular group. For patients with *POLE*mut endometrial cancer, de-escalation of adjuvant treatment will be studied in a registry; for those with mismatch repair deficiency cancers, radiation will be compared with radiation plus checkpoint inhibition; for patients with p53abn cancers, chemoradiation will be compared with chemoradiation followed by PARP inhibition; and for those with no specific molecular profile cancers, adjuvant chemoradiation will be compared with approaches including hormonal treatment. Currently, the molecular classification is increasingly incorporated in the clinical diagnostic pathology. Implementation of the molecular-based classification for all endometrial cancers, or just high-grade tumors, depends on available resources. However, it might well prove to be cost-effective as unnecessary or ineffective adjuvant treatment can be reduced. In the coming years, the molecular classification will become the basis of molecular sub-group directed adjuvant treatment approaches and of new trial designs that explore novel, more individualized targeted treatments. Many studies on (novel) targeted agents and combination treatments as adjuvant treatment or treatment of recurrent or metastatic disease are ongoing. Results of these trials are likely to have profound impact on treatment guidelines in the coming years.
